# Effect of royal jelly ingestion for six months on healthy volunteers

**DOI:** 10.1186/1475-2891-11-77

**Published:** 2012-09-21

**Authors:** Hiroyuki Morita, Takahide Ikeda, Kazuo Kajita, Kei Fujioka, Ichiro Mori, Hideyuki Okada, Yoshihiro Uno, Tatsuo Ishizuka

**Affiliations:** 1 Department of General Internal Medicine, Gifu University Graduate School of Medicine, Gifu, 501-1194, Japan

**Keywords:** Testosterone, Dehydroepiandrosterone sulfate, Erythropoiesis, Glucose tolerance, SF-36

## Abstract

**Background:**

Royal jelly is a widely ingested supplement for health, but its effects on humans are not well known. The objective was to evaluate the effects of long-term royal jelly ingestion on humans.

**Methods:**

We conducted a randomized placebo-controlled, double-blind trial. A total of 61 healthy volunteers aged 42-83 years were enrolled and were randomly divided into a royal jelly group (n = 31) and a control group (n = 30). Three thousand mg of royal jelly (RJ) or a placebo in 100 ml liquid/day were ingested for 6 months. The primary outcomes were changes in anthropometric measurements and biochemical indexes from baseline to 6 months after intervention.

**Results:**

Thirty subjects in the RJ group and 26 in the control group were included in the analysis of endpoints. In an adjusted mean change of the variables from the baseline, significant differences between the two groups could be found in red blood cell counts (+0.16x10^6^ /μL for the RJ group vs. -0.01x10^6^ /μL for the control group, *P* = 0.0134), hematocrit (+0.9% vs. -0.8%, *P* = 0.0251), log (fasting plasma glucose) (+0.01 ± 0.01 log mg/dL vs. +0.05 ± 0.01 log mg/dL, *P* = 0.0297), log (insulinogenic index) (+0.25 vs. -0.13, *P* = 0.0319), log dehydroepiandrosterone sulfate (DHEA-S) (+0.08 log μg/dL vs. +0.20 log μg/dL, P = 0.0483), log testosterone (T) (+0.12 ± 0.04 log ng/mL vs. -0.02 ± 0.05 log ng/mL, *P* = 0.0416), log T/DHEA-S ratio (+0.05 ± 0.05 vs. -0.23 ± 0.59, *P* = 0.0015), and in one of the SF-36 subscale scores, mental health (MH) (+4 vs. -7, *P* = 0.0276).

**Conclusions:**

Six-month ingestion of RJ in humans improved erythropoiesis, glucose tolerance and mental health. Acceleration of conversion from DHEA-S to T by RJ may have been observed among these favorable effects.

## Introduction

Royal jelly (RJ) is mainly secreted by the hypopharyngeal and mandibular glands of worker honeybees (*Apis mellifera*) between the sixth and twelfth days of their life [1] and is an essential food for the development of the queen honeybee. RJ is a complex substance containing a unique combination of proteins (12-15%), sugars (10-12%), lipids (3-7%), amino acids, vitamins, and minerals [2]. Compared with the short-lived and infertile worker bees, the queen bee, which is exclusively fed RJ, is characterized by her extended lifespan and her well-developed gonads. Therefore, RJ has been long- used as a supplement for nutrition, anti-aging or infertility.

RJ has been demonstrated to possess many pharmacological activities in experimental animals, including antitumor [3], anti-oxidant [4], anti-inflammatory [5], antibacterial [6], anti-allergic [7], anti-aging [8] and antihypertensive properties [9]. In humans, its oral ingestion improves lipoprotein metabolism and reduces serum total cholesterol (TC) and low-density lipoprotein (LDL) levels [10]. Lady 4, a combination of four natural components including RJ, promoted health and well-being in postmenopausal women [11].

RJ develops the queen bee gonads. An RJ diet induced higher testosterone (T) content and more intensive spermatogenesis in hamster testis [12] and increased serum testosterone levels in heat stressed male rabbits [13]. It may also modulate sex hormones in humans. Dehydroepiandrosterone sulfate (DHEA-S), which declines during normal aging, may serve as a potential longevity marker [14,15] and may improve insulin resistance [16-18]. Men with higher serum DHEA-S had a longer life span in a Baltimore longitudinal study of aging male humans [19]. Estradiol (E2) is more important than testosterone in the pathway to insulin resistance in healthy, young postmenopausal women [20].

We conducted a randomized placebo-controlled, double-blind trial to evaluate how RJ affects biochemical, nutritional and glucose tolerance.

## Subjects and methods

### Subjects

All subjects were healthy volunteers recruited mainly among adults living in Takayama City in Japan in May 2008. They were users of a home health care telemedicine system managed by ISET Co. (Tsu, Japan). The purpose, benefit and risk of this study were explained to each subject and a written informed consent was obtained from all the subjects. Their lifestyle habits, past history, present illness and medicines were obtained by a written questionnaire at the enrollment. Diabetic patients with poor blood sugar control (HbA1c > = 7.4%) were excluded. A subject who had undertaken a gastrectomy was also excluded.

**Figure 1:**
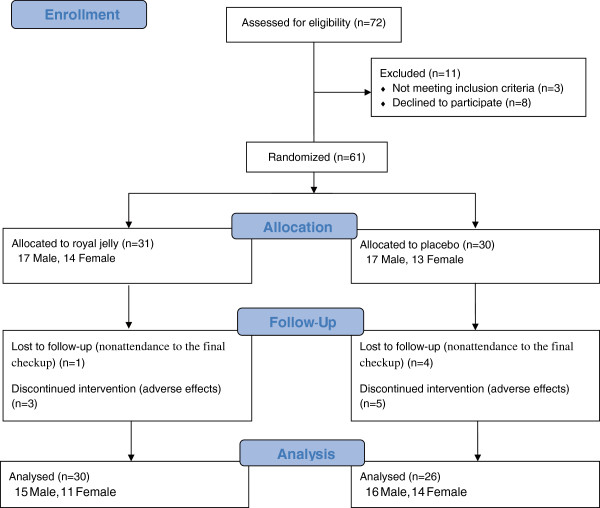
Flow of participants through the present study.

Sixty-one healthy volunteers aged 42–83 years were enrolled in the present study (Figure 1). The subjects were randomly divided into two groups: one was a RJ group (n = 31) and the other was a control group (n = 30). Randomization was performed by random numbers generated by a computer at ISET Co. The RJ group took 100 ml liquid containing 3000 mg of RJ a day for six months and the control group received the same volume and appearance of liquid without containing RJ as a placebo for the same period. The same amount of ingredients, such as fructose, citrate, vitamin B2 and several flavors, were contained in both kinds of liquid. These liquids were prepared by API Co., Ltd. (Gifu, Japan) and were provided to all the participants. The trial was approved by the Ethics Committee of Gifu University Graduate School of Medicine (No. 20–34).

## Methods

Anthropometric measurements, blood and urinary examinations were assessed at a checkup which we held before and after intervention for six months. Height, weight, body mass index (BMI), systolic and diastolic blood pressures, pulse rate and waist circumference were measured as anthropometry. Brachial-ankle pulse wave velocity (baPWV) and bone mineral density (BMD) at the calcaneus were determined using a pulse pressure analyzer (Form PWV/ABI; Nihon Colin Co., Tokyo, Japan) and an ultrasound bone densitometer (CM-200; Erk Co., Tokyo, Japan), respectively. Blood pressure, BMD and baPWV were measured twice and the mean values were adopted.

All blood samples were drawn between 0700 and 0800 h a.m. after an overnight fast. A 75-g oral glucose tolerance test (OGTT) for measuring glucose and insulin drawn before and at 30 and 120 min after oral glucose ingestion was performed. Additional assessments included as follows: screening blood tests, HbA1c, plasma homocystein and 3-methoxy-4-hydroxy-phenylglycol (MHPG), serum cystatin C, high sensitive C-reactive protein (hsCRP), leptin, high-molecular-weight adiponectin (hmw-adiponectin), prolactin, E2, T, DHEA-S, undercarboxylated osteocalcin (ucOC), N-telopeptides of type I collagen (NTx) and ratio of urinary 8-hydroxy-2’-deoxyguanosine (8-OHdG) to creatinine. Plasma and serum was obtained by centrifuging the blood at 1,000 x g for 15 min at 4C immediately after drawing blood. We commissioned SRL Inc. (Tachikawa, Japan) to measure all of the hematological variables, serum and urinary biochemical and hormonal concentrations. Homocystein and MHPG were measured by HPLC, cystatin C by colloidal gold enhanced immunoassay, leptin, E2 and ucOC by RIA, hmw-adiponectin, prolactin, T and DHEA-S by CLEIA, and hsCRP, NTx and 8-OHdG by ELISA.

The homeostatic model assessment-insulin resistance index (HOMA-IR), which is calculated as fasting immunoreactive insulin (FIRI) (μU/mL) x fasting plasma glucose (FPG) (mg/dL) divided by 405, was used to assess insulin resistance. HOMA of β-cell function index (HOMA-B), computed as the product of 360 x FIRI (μU/mL) divided by the value of FPG (mg/dL) minus 63, has been proposed to be a good measure of steady state β-cell function. Insulinogenic index (IGI), which is the ratio of the 30-min increment in insulin level to the 30-min increment in glucose level in the OGTT, was used as an index of early-phase insulin secretion from β-cells. Estimated glomerular filtration rate (eGFR) and non-high density lipoprotein cholesterol (non-HDL-C) were calculated as 194 x age^−0.287^ x serum creatinine (CRE) ^−1.094^ (x 0.739 if female) and TC - HDL-C, respectively.

General health status was assessed with the use of the Japanese version of the Medical Outcomes Study 36-Item Short-Form Health Survey (SF-36v2^TM^) [21,22] before and after intervention. The SF-36, a standardized and written questionnaire, evaluates eight dimensions of health: physical functioning (PF), role limitations due to physical problems (RP), bodily pain (BP), general health perception (GH), vitality (VT), social functioning (SF), role of limitation due to emotional problems (RE) and mental health (MH). Scores for each domain range from 0 to 100. A higher score indicates better health status. The scores were calculated by a Japanese version of the scoring program. The SF-36v2^TM^ and the scoring program were obtained from the Institute for Health Outcomes & Process Evaluation Research (Kyoto, Japan).

A target sample size of 58 participants (29 subjects per group) was estimated to provide > =80% power at a 5% level of significance (2-sided) to detect a 0.9 difference in the change in hematocrit (Ht) from baseline to the end of intervention period between the 2 groups, with an assumption of an SD of 5.0 and allowance for a 10% loss to follow up.

### Statistical analysis

Analyses were done according to intention to treat. Data were expressed as mean ± SEM. Baseline characteristics were compared between the RJ group and the control group using the Fisher exact test for categorical variables and Student’s *t*-test for continuous ones. Non-normally distributed variables were log transformed for further analysis if they were applicable. An analysis of covariance (ANCOVA), adjusted for age, sex, smoking and drinking habits, hypertension, diabetes mellitus, dyslipidemia, arrhythmia, history of ischemic heart disease and apoplexy and the baseline value as covariates, was used to compare change from baseline to 6 months after intervention in anthropometric and biochemical variables and the SF-36 subscale scores between the RJ and control groups. Regarding DHEA-S, T and E2, analyses by gender were also done because the normal range of these hormones was quite different between the sexes. *P* values less than 0.05 were considered statistically significant. SAS 9.1.3 Service Pack 4 for Windows (SAS Institute Inc., Cary, NC) was used for all statistical analyses.

## Results

Among the initially enrolled 61 volunteers, 3 in the RJ group and 5 subjects in the control group quit the ingestion during the intervention period (Figure 1). The reasons why they gave it up were abdominal fullness, diarrhea, and dislike of the taste but the symptoms were not severe and they recovered after discontinuance. There was no difference in the adverse effects between the control and RJ groups. Among the 7 subjects who gave up the ingestion, two in the RJ group and two in the control group underwent the final checkup and were not excluded from the present study according to intention to treat analysis. One subject in the control group who ingested the placebo completely for six months was excluded because of nonattendance to the final checkup. Finally, 30 in the RJ group and 26 subjects in the control group were analyzed.

There were no significant differences in age, sex, smoking and drinking habits, hypertension, diabetes mellitus, dyslipidemia, arrhythmia, history of ischemic heart disease and stroke at baseline between the two groups (data not shown). Among the anthropometric and biochemical examinations, all variables were no different between the groups except for lower PF and MH of SF-36 subscales in the RJ group as shown in Table 1.

**Table 1:** Main baseline characteristics of the subjects in the RJ and control group

	**RJ**	**Control**	***P *value**
BMI (kg/m^2^)	22.7±0.5	22.8±0.5	0.9533
Waist circumference (cm)	86.3±1.3	84.1±1.5	0.2626
Systolic BP (mmHg)	135±3	137±4	0.7135
Diastolic BP (mmHg)	79±2	80±2	0.6125
WBC (/μL)	5090±240	5420±280	0.3652
RBC (x10^6^/μL)	4.52±0.08	4.64±0.08	0.3049
Hb (g/dL)	14,2±0.3	14.5±0.3	0.5076
Ht (%)	44.6±0.7	46.0±0.9	0.1915
PLT (x10^3^/μL)	193±11	211±10	0.2222
log TG (log mg/dL)	4.79±0.13	4.54±0.09	0.1128
HDL-C (mg/dL)	57±3	59±3	0.5709
LDL-C (mg/dL)	110±5	126±4	0.0186
log FPG (log mg/dL)	4.62±0.03	4.63±0.02	0.7231
log FIRI (log μIU/mL)	1.38±0.14	1.66±0.11	0.1375
log HbA1c (log %)	1.7±0.0	1.7±0.0	0.6786
log HOMA-IR	-0.00±0.16	0.29±0.11	0.1399
log HOMA-B	3.66±0.13	3.90±0.12	0.2009
log IGI	-0.54±0.18	-0.41±0.20	0.6309
log DHEA-S (log μg/dL)	4.22±0.10	4.46±0.13	0.1541
male	4.52±0.11	4.74±0.13	0.2044
female	3.88±0.20	4.07±0.19	0.4258
log T (log ng/mL)	-0.32±0.38	0.01±0.41	0.5544
male	1.52±0.05	1.68±0.08	0.0926
female	-2.41±0.25	-2.27±0.25	0.6829
log E2 (log pg/mL)	2.41±0.14	2.74±0.21	0.1948
male	3.11±0.08	3.29±0.09	0.1138
female	1.61±0.00	1.99±0.38	0.3409
log T/DHEAS	-6.84±0.32	-6.74±0.34	0.8436
male	-5.30±0.11	-5.35±0.16	0.7915
female	-8.59±0.20	-8.64±0.13	0.8389
log E2/T	-4.18±0.26	-4.18±0.30	0.9994
male	-5.32±0.08	-5.30±0.08	0.8960
female	-2.88±0.25	-2.65±0.31	0.5678
SF-36 subscale scores			
PF	81±3	89±2	0.0393*
RP	84±4	88±4	0.4342
BP	66±5	69±4	0.5467
GH	61±3	63±3	0.5936
VT	61±3	67±3	0.1529
SF	88±3	94±2	0.0922
RE	91±3	91±3	0.9693
MH	71±3	80±3	0.0289*

Abbreviations were denoted in the text. * *P* < 0.05.

In the mean change of variables from the baseline adjusted for age, sex, smoking and drinking habits, hypertension, diabetes mellitus, dyslipidemia, arrhythmia, history of ischemic heart disease and apoplexy, peripheral red blood cell counts (RBC), Ht, log IGI, log T, log T/DHEA-S ratio and MH of SF-36 subscales were higher and log FPG, and log DHEA-S were lower in the RJ group than those in the control group (Table 2). According to analysis by gender, the change of log T/DHEA-S ratio in men was significantly higher in the RJ group but that of log E2/T in both sexes was no different between the groups. NTx, PF and MH were higher in men of the RJ group than in those in the control one (data not shown). Log TG and Ht were higher and log FPG was lower in women (data not shown).

**Table 2:** Changes of main variables from baseline to 6 months after intervention

	**RJ**	**Control**	***P *value**
BMI (kg/m^2^)	+0.1±0.2	+0.0±0.2	0.8389
Waist circumference (cm)	-2.1±0.6	-2.0±0.7	0.9115
Systolic BP (mmHg)	-0±3	-1±3	0.8732
Diastolic BP (mmHg)	+0±2	-1±2	0.6321
WBC (/μL)	+570±270	+320±300	0.5661
RBC (x10^6^/μL)	+0.16±0.04	-0.01±0.05	0.0134*
Hb (g/dL)	+0.8±0.2	+0.3±0.2	0.0548
Ht (%)	+0.9±0.5	-0.8±0.5	0.0251*
PLT (x10^3^/μL)	+1±+1	+1±1	0.5809
log TG (log mg/dL)	+0.04±0.06	-0.05±0.07	0.3323
HDL-C (mg/dL)	+2±2	-1±2	0.2586
LDL-C (mg/dL)	+9±3	+4±3	0.2690
log FPG (log mg/dL)	+0.01±0.01	+0.05±0.01	0.0297*
log FIRI (log μIU/mL)	+0.05±0.11	+0.04±0.12	0.9486
log HbA1c (log %)	-0.0±0.0	-0.0±0.0	0.9945
log HOMA-IR	+0.07±0.11	+0.08±0.13	0.9296
log HOMA-B	+0.02±0.10	-0.05±0.11	0.6027
log IGI	+0.25±0.11	-0.13±0.12	0.0319*
log DHEA-S (log μg/dL)	+0.08±0.04	+0.20±0.04	0.0483*
male	+0.06±0.06	+0.22±0.07	0.1517
female	+0.11±0.04	+0.17±0.05	0.4672
log T (log ng/mL)	+0.12±0.04	-0.02±0.05	0.0416
male	+0.06±0.03	-0.06±0.03	0.0503
female	+0.24±0.10	-0.02±0.11	0.1369
log E2 (log pg/mL)	-0.01±0.04	+0.04±0.04	0.3955
male	+0.02±0.06	+0.07±0.06	0.6303
female	-0.06±0.06	+0.03±0.07	0.3756
log T/DHEAS	+0.05±0.05	-0.23±0.59	0.0015*
male	-0.01±0.06	-0.27±0.06	0.0153*
female	+0.12±0.10	-0.19±0.12	0.0848
log E2/T	-0.11±0.06	+0.04±0.06	0.1034
male	+0.01±0.07	+0.09±0.07	0.4745
female	-0.28±0.11	+0.02±0.13	0.1353
SF-36 subscale scores			
PF	+2±1	-1±1	0.1384
RP	-6±3	-4±3	0.6050
BP	+0±4	-4±4	0.4701
GH	+16±12	-6±14	0.2856
VT	+7±3	-1±3	0.0924
SF	-1±4	-6±4	0.4395
RE	-3±3	-4±3	0.8287
MH	+4±3	-7±3	0.0276*

Abbreviations were denoted in the text. * *P* < 0.05.

There were no significant differences in BMI, waist circumference, lipids, hepatic and renal functions, atherosclerotic (blood pressure, baPWV, homocystein and hmw-adiponectin), other glycemic (log HbA1c, log HOMA-IR, log HOMA-B and log leptin), and bone metabolic variables (BMD, NTx, log ucOC) (data not shown).

## Discussion

The honeybee forms two female castes: the queen and the worker. This dimorphism does not depend on genetic differences but on ingestion of RJ. Recently, it has been demonstrated that royalactin, a 57-kDa protein, in RJ induces the differentiation of honeybee larvae into queens through an epidermal growth factor receptor-mediated signaling pathway, increasing body size and ovary development [23].

RJ has been widely used as a supplemental food for health promotions, but little effects on human beings have been objectively shown. Only three randomized controlled trials to investigate the effect of RJ on humans have been published in English so far. Improvement of lipid metabolism [10], quality of life in postmenopausal women [11] and fertility of couples affected by asthenozoospermia [24] have been reported.

The present trail has shown increases in RBC and Ht as well as improvement of glucose tolerance and mental health. However, no apparent effect on serum lipids as shown by Guo *et al.*[10] was observed. They have shown that serum TC and LDL-C in the RJ group decreased significantly more than those in the control group. One of the reasons why we could not obtain any effect on serum lipids is smaller doses of RJ in our study. The subjects in their RJ group had taken 6 g of RJ a day for 4 weeks, in contrast to 3000 mg a day for 6 months in our RJ trial. Another possibility is that the lipid lowering effect of RJ may be transient and not last for six months.

We could not find any papers which described the effect of RJ on anemia. Most of our participants did not have anemia and the increase in RBC and Ht was modest. There was no significant change in mean corpuscular volume and mean corpuscular hemoglobin between before and after intervention (data not shown), suggesting that RJ did not promote iron metabolism or hemoglobin synthesis but stimulated erythropoiesis or prolonged the lifespan of erythrocytes. The most probable reason is acceleration of erythropoiesis by testosterone which increased in serum in the present study. Testosterone is an anabolic steroid and has been used to treat several types of anemia.

**Figure 2:**
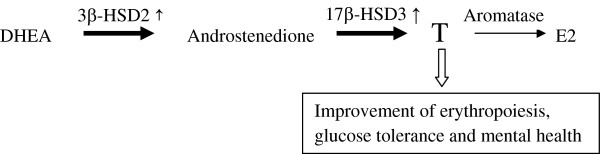
Acceleration of conversion from DHEA to T by six-month ingestion of RJ, resulting in improvement of erythropoiesis, glucose tolerance and mental health.

E2 is a primary estrogen in humans and is converted from T by aromatase located in the ovary or adipose tissues. Most of our female participants were over the age of 50 and after menopause, resulting in lower serum E2 levels in women than those in men. DHEA-S is the most abundant androgen in human beings and is synthesized in the adrenal. It is then converted to T through androstenedione by 3β-hydroxysteroid dehydrogenase type 2 (3β-HSD2) and 17β-hydroxysteroid dehydrogenase type 3 (17β-HSD3) located in the adrenal and testis [25]. In the present trial, less increase in DHEA-S and more increase in T in the RJ group than in the control one was observed, suggesting that RJ may have induced the conversion of DHEA-S to T by stimulating 3β-HSD2 and/or 17β-HSD3 as shown in Figure 2. Serum T/DHEA-S and E2/T ratios are considered to be designated activities of 3β-HSD2 and/or 17β-HSD3 and aromatase, respectively. The changes of log T/DHEA-S ratio were significantly higher only in the men, suggesting that RJ may induce 3β-HSD2 and/or 17β-HSD3 activity in the testis. It is possible that RJ also could promote those enzyme activities in the ovary if the women had normal menstrual cycles. In contrast, the changes of log E2 and log E2/T ratios were not different, indicating that RJ may have no action on aromatase in human beings. The enhanced 3β-HSD2 and/or 17β-HSD3 activity may result from high antioxidant enzyme activities of RJ [26].

RJ did not improve HOMA-IR or HbA1c but FPG and IGI were in the present study, suggesting that RJ stimulated insulin secretion. The improvement of glucose tolerance may have been brought about by a rise of T. T replacement restores insulin action, increases islet insulin content, and enhances insulin secretion in rats [27]. In contrast, treatment with T in elderly men does not improve carbohydrate tolerance or alter insulin secretion, insulin action, or glucose effectiveness [28].

No significant differences in atherosclerotic and bone metabolic variables were observed (data not shown). The six month intervention may have been too short to elucidate the effect on baPWV and BMD. However, no effects on other variables, homocystein, hmw-adiponectin, NTx and ucOC, may indicate that RJ has no beneficial potency on atherosclerosis or bone metabolism.

Improvement of MH of SF-36 subscales was observed. The changes of MH as well as PF were also significant in men, suggesting that the beneficial effects on quality of life in men may be a secondary action of T elevation. In late onset hypogonadism patients, testosterone replacement therapy resulted in the improvement of six of eight domains including MH and PF in SF-36 [29]. In a stress-inducible depression-like mouse model, 10-hydroxy-trans-2-decenoic acid, an unsaturated fatty acid unique to RJ, protected against depression and anxiety [30].

In the present study, we did not observe any serious adverse effects of RJ. In the literature, several cases of hemorrhagic colitis [31], asthma [32] and anaphylaxis [33] by ingestion of RJ have been reported. RJ should be considered as a causative allergen. Increased consumption of RJ in health food supplements may increase incidence of RJ-related allergic reactions [33].

### Limitations

This study had several limitations. First, the sample size was minimal and may have been too small for some measures to reach statistical significance, especially in the case of analysis by gender. Second, we do not know the most effective dose of RJ for a human being. Larger or smaller daily amounts of RJ may have been necessary to produce some important effects.

## Conclusion

In conclusion, six-month ingestion of RJ in humans improved erythropoiesis, glucose tolerance and mental health. These may be due to secondary effects of T with acceleration of conversion from DHEA-S to T by activation of 3β-HSD2 and/or 17β-HSD3 through anti-oxidant enzyme potential of RJ. A RCT with larger number of subjects will be necessary to further verify the effects of RJ in more detail.

## Abbreviations

RJ: Royal jelly; DHEA-S: Dehydroepiandrosterone sulfate; T: Testosterone; TC: Total cholesterol; LDL: Low density lipoprotein; E2: Estradiol; HbA1c: Hemoglobin A1c; BMI: Body mass index; baPWV: Brachial-ankle pulse wave velocity; BMD: Bone mineral density; OGTT: 75 g oral glucose tolerance test; MHPG: 3-methoxy-4-hydroxy-phenylglycol; hsCRP: High sensitive C-reactive protein; hmw: High-molecular-weight; ucOC: Undercarboxylated osteocalcin; NTx: N-telopeptides of type I collagen; 8-OHdG: 8-hydroxy-2’-deoxyguanosine; HOMA-IR: Homeostatic model assessment-insulin resistance index; FIRI: Fasting immunoreactive insulin; FPG: Fasting plasma glucose; HOMA-B: HOMA of β-cell function index; IGI: Insulinogenic index; eGFR: Estimated glomerular filtration rate; HDL-C: High density lipoprotein cholesterol; CRE: Creatinine; SF-36: Medical Outcomes Study 36-Item Short-Form Health Survey; PF: Physical functioning; RP: Role limitations due to physical problems; BP: Bodily pain; GH: General health perception; VT: Vitality; SF: Social functioning; RE: Role of limitation due to emotional problems; MH: Mental health; RBC: Red blood cell counts; Ht: Hematocrit; RIA: Radioimmunoassay; CLEIA: Chemiluminescent enzyme immunoassay; ELISA: Enzyme-linked immunosorbent assay; ANCOVA: Analysis of covariance; 3β-HSD2: 3β-hydroxysteroid dehydrogenase type 2; 17β-HSD3: 17β-hydroxysteroid dehydrogenase type 3.

## Competing interests

The authors declare no conflict of interest.

## Authors' contributions

The authors’ responsibilities were as follows: HM, KK and TIs designed and conducted research; HM, KK, TIk, IM, KF, HO and YU participated in the checkups and collected data; HM and YU performed the statistical analysis; HM and TIs wrote the paper; HM had primary responsibility for final content. All authors read and approved the final manuscript. The study sponsors had no role in the study design; collection, analysis, or interpretation of the data; writing of the paper; or decision to submit the manuscript for publication.
